# Diagnostic performances of GPT-4o, Claude 3 Opus, and Gemini 1.5 Pro in “Diagnosis Please” cases

**DOI:** 10.1007/s11604-024-01619-y

**Published:** 2024-07-01

**Authors:** Yuki Sonoda, Ryo Kurokawa, Yuta Nakamura, Jun Kanzawa, Mariko Kurokawa, Yuji Ohizumi, Wataru Gonoi, Osamu Abe

**Affiliations:** Department of Radiology, Graduate School of Medicine, The University of Tokyo, 7-3-1 Hongo, Bunkyo-ku, Tokyo, 113-8655 Japan

**Keywords:** Large language model, Artificial intelligence, ChatGPT, GPT-4o, Claude 3 opus, Gemini 1.5 pro

## Abstract

**Purpose:**

Large language models (LLMs) are rapidly advancing and demonstrating high performance in understanding textual information, suggesting potential applications in interpreting patient histories and documented imaging findings. As LLMs continue to improve, their diagnostic abilities are expected to be enhanced further. However, there is a lack of comprehensive comparisons between LLMs from different manufacturers. In this study, we aimed to test the diagnostic performance of the three latest major LLMs (GPT-4o, Claude 3 Opus, and Gemini 1.5 Pro) using Radiology Diagnosis Please Cases, a monthly diagnostic quiz series for radiology experts.

**Materials and methods:**

Clinical history and imaging findings, provided textually by the case submitters, were extracted from 324 quiz questions originating from Radiology Diagnosis Please cases published between 1998 and 2023. The top three differential diagnoses were generated by GPT-4o, Claude 3 Opus, and Gemini 1.5 Pro, using their respective application programming interfaces. A comparative analysis of diagnostic performance among these three LLMs was conducted using Cochrane’s Q and post hoc McNemar’s tests.

**Results:**

The respective diagnostic accuracies of GPT-4o, Claude 3 Opus, and Gemini 1.5 Pro for primary diagnosis were 41.0%, 54.0%, and 33.9%, which further improved to 49.4%, 62.0%, and 41.0%, when considering the accuracy of any of the top three differential diagnoses. Significant differences in the diagnostic performance were observed among all pairs of models.

**Conclusion:**

Claude 3 Opus outperformed GPT-4o and Gemini 1.5 Pro in solving radiology quiz cases. These models appear capable of assisting radiologists when supplied with accurate evaluations and worded descriptions of imaging findings.

## Introduction

Large language models (LLMs) are neural network models trained on vast amounts of text data that demonstrate high performance in natural language processing tasks [1]. They are used in various fields, including medicine [2].

The application of LLMs in the field of radiology has also been discussed [3, 4], and previous studies have investigated the diagnostic capabilities of LLMs in radiology. In a study by Ueda et al. [5], OpenAI’s GPT-4 model [6] achieved a 54% accuracy rate, correctly answering 170 out of 313 cases in “Diagnosis Please,” a monthly diagnostic radiology quiz case series for radiology experts published in the international academic journal Radiology. The model relied solely on the clinical history and imaging findings provided for each case. Similarly, Horiuchi et al. [7] reported that the GPT-4 model achieved a diagnostic accuracy of 50% (50/100 cases) in “Case of the Week,” a diagnostic quiz case series published in the American Journal of Neuroradiology, also based on the provided clinical history and imaging findings. A study by Toyama et al. [8] evaluated the performance of LLMs on the Japanese Radiological Society board exam, demonstrating the differences in performance among LLMs and their ability to handle radiological tasks in languages other than English.

Recently, various manufacturers developed LLMs that have undergone multiple version upgrades. The flagship models include OpenAI’s GPT-4o model [9], Anthropic’s Claude 3 Opus [10], and Google’s Gemini 1.5 Pro [11]. Li et al. [12] demonstrated a significant improvement in diagnostic performance using the GPT-4 model compared with the GPT-3.5 Turbo model. This suggests that version upgrades can indeed improve the diagnostic performance of LLMs.

In a previous study, we evaluated the diagnostic performance of Claude 3 Opus using Diagnosis Please cases and reported a diagnostic accuracy of 62.1% for the top three differential diagnoses based on textual clinical history and imaging findings [13]. However, the diagnostic abilities of the recently released GPT-4o and Gemini 1.5 Pro have not been investigated.

Therefore, in this study, we aimed to assess and compare the diagnostic performance of three flagship models—GPT-4o, Claude 3 Opus, and Gemini 1.5 Pro—on Diagnosis Please cases using clinical history and imaging findings. Our goal is to provide insights into the diagnostic capabilities of the current state-of-the-art LLMs and highlight the potential differences between these advanced models.

## Materials and methods

**Fig. 1:**
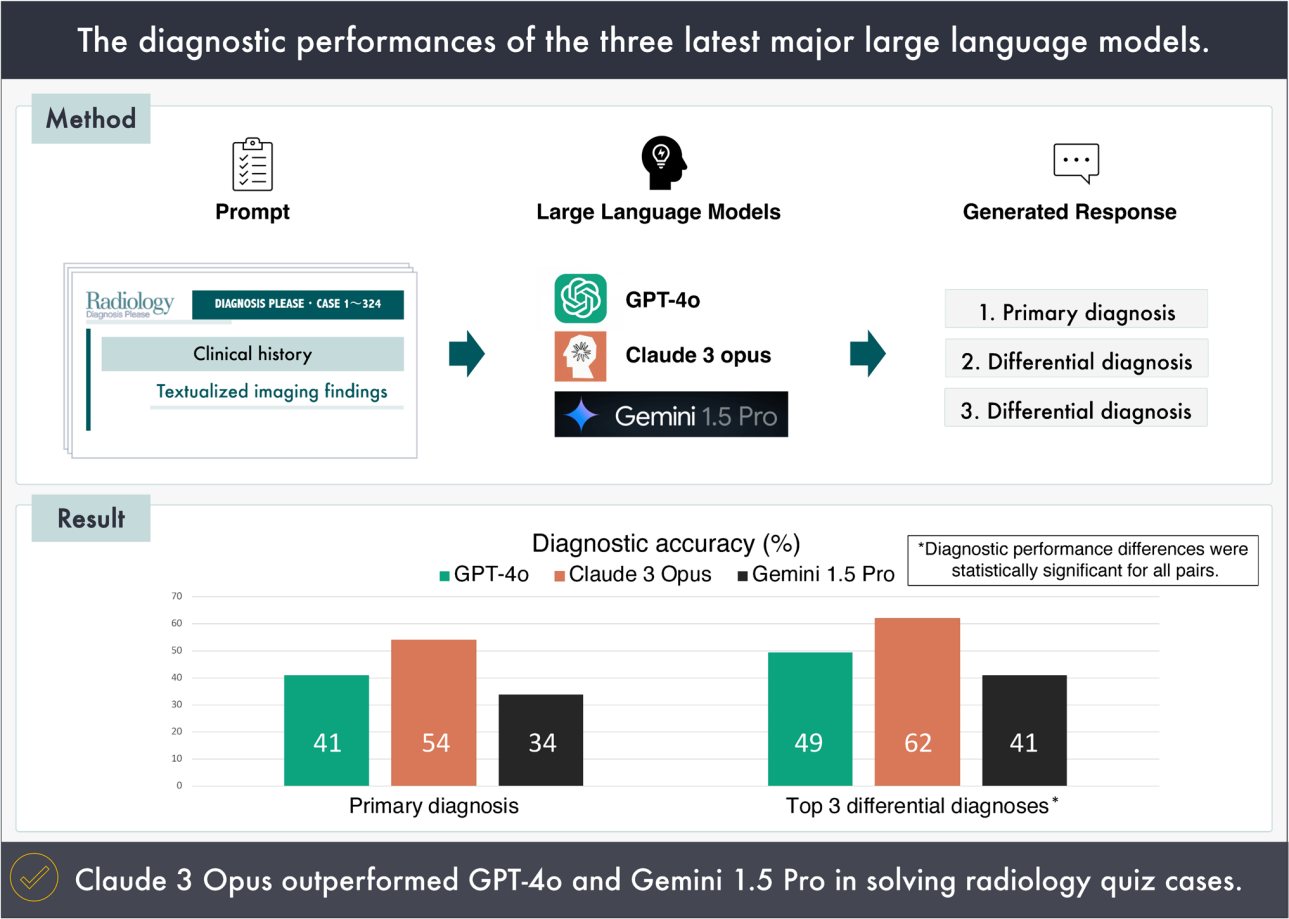
Overview of the present study

An overview of this study is presented in Fig. 1.

We used GPT-4o (OpenAI, San Francisco, United States; released on May 13, 2024), Claude 3 Opus (Anthropic, California, United States; released on March 4, 2024), and Gemini 1.5 Pro (Google, Mountain View, United States; released on April 9, 2024) to list the primary diagnoses and two differential diagnoses for the 324 quiz questions (cases 1–324, published between 1998 and 2023) from Radiology Diagnosis Please (https://dxp.rsna.org/).

Application programming interfaces (APIs) were used to access each model (GPT-4o: gpt-4o-2024-05-13, Claude 3 Opus: claude-3-opus-20240229, and Gemini 1.5 Pro: gemini-1.5-pro-latest) on May 18, 2024. To ensure reproducibility, we specified the generation parameters for all models as temperature = 0.0 and top-p = 1.0. To prevent previous inputs from influencing subsequent ones, inputs were conducted in an independent session for each case. The prompt was as follows [7]: “As a physician, I plan to utilize you for research purposes. Assuming you are a hypothetical physician, please walk me through the process from differential diagnosis to the most likely diagnosis and the next two most likely differential diagnoses step-by-step based on the attached patient's information.”

**Fig. 2:**
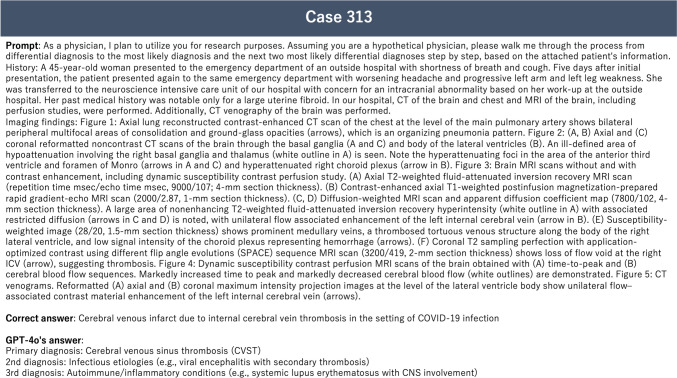
Correct answer and GPT-4o generated response for Radiology Diagnosis Please Case 313 [14]. Based on the provided case description and chest CT findings, it can be inferred that COVID-19 acted as a trigger for cerebral vein thrombosis. The response generated by GPT-4o failed to explicitly mention the potential causal relationship between COVID-19 and CVST, which is a crucial aspect of this case. Consequently, the response was deemed to lack sufficient elements and was judged as incorrect

Each prompt was submitted to the LLMs only once, and the first response generated was used for evaluation. When extracting the submitter-identified imaging findings, one trainee radiologist and one board-certified diagnostic radiologist with 11 years of experience meticulously removed sentences containing answers to ensure analytical integrity. The accuracy of the LLMs' primary diagnosis and the two differential diagnoses was determined by consensus of three board-certified diagnostic radiologists with respective experience of 8, 11, and 19 years. Responses deemed ambiguous or lacking sufficient elements were categorized as incorrect (Fig. 2) [14].

Given that this study relied on previously published articles, ethical approval was deemed unnecessary.

The Cochrane *Q* test was used to assess the differences in performance among the three LLMs. When significant differences were detected, post hoc analyses were conducted using McNemar’s tests with continuity correction and Bonferroni’s correction to evaluate the differences in accuracy rates for the top three differential diagnoses between each pair of models. Two-sided *p* values < 0.05 were considered statistically significant. Statistical analyses were performed using the R software (version 4.1.1; R Foundation for Statistical Computing, Vienna, Austria).

## Results

The diagnostic accuracies for primary diagnosis were 41.0%, 54.0%, and 33.9% for GPT-4o, Claude 3 Opus, and Gemini 1.5 Pro, respectively. These accuracy rates increased to 49.4%, 62.0%, and 41.0% for GPT-4o, Claude 3 Opus, and Gemini 1.5 Pro, respectively, when the top three differential diagnoses were considered. Notably, Gemini 1.5 Pro responded with "Providing a differential diagnosis based on the information provided would be irresponsible and potentially harmful” for six out of 324 questions, which was deemed an incorrect answer. Except for the above six answers, the LLMs provided three differential diagnoses for all the other questions.

The Cochrane Q test demonstrated significant differences in diagnostic performance among the three LLMs (*p* < 0.001). Post hoc pairwise comparisons using McNemar's tests with continuity correction and Bonferroni's correction revealed that Claude 3 Opus outperformed GPT-4o (*p* < 0.001), which, in turn, outperformed Gemini 1.5 Pro (*p* = 0.001). Significant differences were observed among all the combinations of LLMs (Table 1).

**Table 1:** Diagnostic accuracy of each model, Cochrane’s Q test, and McNemar’s tests between each model

	Accuracy			Cochrane’s Q test	McNemar’s test		
	GPT-4o	Claude 3 Opus	Gemini 1.5 Pro		GPT-4o vs Claude 3 Opus	GPT-4o vs Gemini 1.5 Pro	Claude 3 Opus vs Gemini 1.5 Pro
Primary diagnosis	41.0% (133/324)	54.0% (175/324)	33.9% (109/324)				
Top 3 differential diagnoses	49.4% (160/324)	62.0% (201/324)	41.0% (132/324)	*p* < 0.001	*p* < 0.001	*p* = 0.001	*p* < 0.001

## Discussion

In this study, we compared the diagnostic performance of flagship LLMs from three companies based on *Radiology* Diagnosis Please cases. To ensure reproducibility and to compare different vendors' LLMs under as similar conditions as possible, we utilized their respective APIs and specified similar parameters for each model. The LLMs were provided with the clinical history and imaging findings from each case.

The results showed that the models performed in the following order from best to worst: Claude 3 Opus, GPT-4o, and Gemini 1.5 Pro. Furthermore, statistically significant differences were observed between all pairwise combinations.

Notably, as of the time of writing, the technical report for GPT-4o has not been released. However, Claude 3 Opus reportedly outperforms Gemini 1.5 Pro on eight text-based language benchmarks, including reasoning, coding, and mathematics [15].

In the context of medical natural language processing capabilities, despite being a general-purpose LLM, Claude 3 Opus achieved an accuracy of 74.9% for 0-shot and 75.8% for 5-shot on PubMedQA [16], which is nearly equivalent to the performance of Google's Med-PaLM 2 [17], an LLM specialized in medicine.

Regarding Gemini 1.5 Pro, one of its design philosophies is the extension of context length [11]. The developers have also released Gemini 1.5 Flash, a lightweight and fast model with slightly reduced performance [15]. These points suggest that the Gemini 1.5 series may prioritize real-world implementations, such as integration into devices, over benchmark performances.

In this study, the accuracy of GPT-4o was lower than that of GPT-4 reported by Ueda et al. [5]. One possible reason for this discrepancy is the strict grading criteria used in this study. This issue arises from the fact that the actual correct answer criteria for Radiology Diagnosis Please cases are not publicly available, which represents a limitation of this study.

Another limitation of this study is potential data leakage. The answers for each case used in this study are available online. According to a previous study on GPT-4 [5], there was no significant difference in the accuracy rate between questions related to the period used for GPT-4's training and those related to the period outside the training. However, considering the vast amount of data these models are trained on, it is possible that some information from these cases was inadvertently included in their training data, which could lead to an overestimation of the LLMs' performance in this study.

In previous studies in which the GPT-4 Turbo with Vision was tasked with solving the Japanese Board of Radiology examination [18], and “Freiburg Neuropathology Case Conference” cases from the journal Clinical Neuroradiology [19], the GPT-4 Turbo with Vision, given both image and textual information, could not outperform the GPT-4 Turbo, which was only provided with textual information. Claude 3 Opus, which achieved the best performance in this study, showed significantly inferior diagnostic performance when given only the history and key images as input, without the textual information of imaging findings, compared to when the textual information of both history and imaging findings were provided, as reported in previous research [13].

In conclusion, at least at present, the main role of LLMs is not to replace radiologists, but rather to assist in diagnosis using imaging findings based on accurate interpretations and verbalization of imaging findings by radiologists.

However, to effectively utilize rapidly evolving LLMs in the field of diagnostic radiology and maximize their potential benefits, it is essential to continue conducting research and evaluations in future. As these models advance and new capabilities emerge, ongoing studies will be crucial to understand their strengths, limitations, and optimal applications in clinical practice.

## Abbreviations

AI: Artificial intelligence
LLM: Large language model
API: Application programming interface
